# Editorial: Resource utilization of agricultural waste through bioprocess engineering for environmental sustainability

**DOI:** 10.3389/fbioe.2023.1147748

**Published:** 2023-03-30

**Authors:** Junting Pan, Yeqing Li, Benyamin Khoshnevisan

**Affiliations:** ^1^ Institute of Agricultural Resources and Regional Planning, Chinese Academy of Agricultural Sciences, Beijing, China; ^2^ State Key Laboratory of Heavy Oil Processing, Beijing Key Laboratory of Biogas Upgrading Utilization, College of New Energy and Materials, China University of Petroleum Beijing (CUPB), Beijing, China; ^3^ Institute of Chemical Engineering, Biotechnology and Environmental Technology, University of Southern Denmark, Odense, Denmark

**Keywords:** agricultural waste, bioconversation, high efficiency, anaerobic digestion (AD), composting

The purpose of this paper is to put together an overview of the recent development of bioconversion processes and their related technology. Its scope is wide so as to cover various aspects of the field, including the high-value conversion of agricultural waste, gas emission reduction, and agricultural waste utilization. Eleven articles were accepted and published in 2022.

Ai et al., from Henan Agricultural University, proposed a method of immobilization of sodium alginate to prepare “slurry capsules” for fermentative biohydrogen production. Not only does this method make biohydrogen slurry a harmless treatment, but it also addresses the problem of producing clean hydrogen energy. Shang et al. team found a strong positive correlation between the increase of N_2_O concentration in biological filter and the elimination ability of NH_3_. According to the authors, reducing the retention time of empty beds could help to prevent greenhouse gases from being produced by biofilters. Gao et al. and his team conducted a comprehensive evaluation of large-scale, multi-component and aerobic composting of rural refuse, which is an extension of the optimization of the previous multi-component mixed compost theory. The co-composting method proposed in this study is strong operable, complies with product standards, and aids in the reduction of waste and the rational utilization of resources. Wang et al., from Yunnan Normal University, used steel slag as a raw material to synthesize a hydrothermally carbonized steel slag. This method innovatively used solid waste-derived environmentally functional materials for heavy metal remediation. This material showed good removal efficiencies for Hg^2+^ and Cr_2_O_7_^2−^. Shi et al. studied the characteristics and bacterial communities of static compost in low temperature and cold areas. Their results suggest that early spring or late fall is suitable for composting and, interestingly, amino acid and carbohydrate metabolism function genes indicate the changes of nitrogen and organic acids. Li et al. studied the effect of heat pre-treatment on the thermophilic anaerobic digestion of high-solid manure. The result shows that the increase of heat treatment biogas could reasonably cover the energy consumption of the pre-treatment itself, and therefore, combining heat pre-treatment with the high-load anaerobic digestion of pig manure is appropriate. Chen et al., through field experiments, provided ideas for improving the soil micro-ecology environment and improving the yield and chemical quality of tobacco. Luo et al. prepared modified biochar from agricultural and industrial by-products. It was found that their modified prepared biochar is a feasible candidate and has a good effect on the removal of selenite from wastewater. Zhang et al. described a variety of technical methods for the improvement of *Saccharomyces cerevisiae* and their industrial applications. The methods to improve the cell surface display efficiency were also discussed. Zhou et al. studied the influence of inorganic and organic fertilizers on crop yield, fauna feeding efficiency, and soil quality. The experimental results suggested that the combination of inorganic fertilizer and organic fertilizer had good effects, such as improving the activity of the soil organic carbon and enzymes and improving the efficiency of the feeding of animals. Heredia Salgado et al. evaluated the transformation of agricultural residues from quinoa and lupin into biochar for soil improvement.

Eleven articles have been published in the journal, which cover a wide range of areas in waste valorization, including garbage, steel slag, and animal feces. The design field is diverse, including clean energy production, reactor research, and the development of biofuel production. This Research Topic may provide new solutions and ideas for future research.

